# Age-Related Increased Onset and Progression of Prostate Cancer Is Revealed in Novel Pten-Null Mouse Models

**DOI:** 10.1093/geroni/igaa057.425

**Published:** 2020-12-16

**Authors:** Qiuyang Zhang, Sen Liu, Bing Zhang, Asim Abdel-Mageed, Chad Steele, Alun Wang, S Jazwinski, Leann Myers

**Affiliations:** 1 Tulane University Schoole of Medicine, New Orleans, Louisiana, United States; 2 Tulane University School of Medicine, New Orleans, Louisiana, United States; 3 Tulane University School of Medicien, New Orleans, Louisiana, United States; 4 Tulane University School of Public Health and Tropical Medicine, New Orleans, Louisiana, United States

## Abstract

Prostate cancer (PCa) is associated with advanced age. To better understand how age impacts PCa, it is critical to use PCa animal models generated at different ages (aged vs. non-aged). The PB-Cre4 driven phosphatase and tensin homolog (Pten) conditional knockout mouse model, which closely imitates human PCa initiation and progression. However, the Pten deletion is triggered in a 2-week-old prostate, when comparing the extent of PCa between aged and non-aged mice, it is difficult to distinguish the extent to which the onset and progression of PCa are due to the acceleration of the normal aging process or due to the manifestation of PCa pathologies over time. We present here a protocol to inject Cre-expressing adenovirus with luciferin tag intraductally into the prostate anterior lobes of Pten floxed mice, thus, the Pten-loss will be triggered at different ages post-Cre expression. The in vivo imaging of luciferin signals following viral infection was conducted to confirm the Cre expression and activity. Immunohistochemical staining was performed to confirm the Cre expression, Pten loss, and p-Akt and p-S6 activation. Prostate weight and histopathology were compared between aged and non-aged mice. The results showed that the virus infection was limited in the prostate glands and aged mice had significantly increased PCa onset and progression compared to young mice. Although technical skill is required to carry out this procedure and the success rate of viral infection is about 80%, this model of PCa is of great use to all investigators in the aging and cancer research field.

